# A Novel Approach to the Surgical Treatment of Lumbar Disc Herniations: Indications of Simple Discectomy and Posterior Transpedicular Dynamic Stabilization Based on Carragee Classification

**DOI:** 10.1155/2013/270565

**Published:** 2013-04-09

**Authors:** A. F. Ozer, F. Keskin, T. Oktenoglu, T. Suzer, Y. Ataker, C. Gomleksiz, M. Sasani

**Affiliations:** ^1^Neurosurgery Department, Koç University School of Medicine, Rumelifeneri Yolu Sarıyer, 34450 Istanbul, Turkey; ^2^Neurosurgery Department, Necmettin Erbakan University Meram Medical Faculty Hospital, 42080 Konya, Turkey; ^3^Neurosurgery Department, American Hospital, 34365 Istanbul, Turkey; ^4^Physical Therapy and Rehabilitation Department, American Hospital, 34365 Istanbul, Turkey; ^5^Neurosurgery Department, Mengücek Gazi Training and Research Hospital, School of Medicine, Erzincan University, 24100 Erzincan, Turkey

## Abstract

Surgery of lumbar disc herniation is still a problem since Mixter and Barr. Main trouble is dissatisfaction after the operation. Today there is a debate on surgical or conservative treatment despite spending great effort to provide patients with satisfaction. The main problem is segmental instability, and the minimally invasive approach via microscope or endoscope is not necessarily appropriate solution for all cases. Microsurgery or endoscopy would be appropriate for the treatment of Carragee type I and type III herniations. On the other hand in Carragee type II and type IV herniations that are prone to develop recurrent disc herniation and segmental instability, the minimal invasive techniques might be insufficient to achieve satisfactory results. The posterior transpedicular dynamic stabilization method might be a good solution to prevent or diminish the recurrent disc herniation and development of segmental instability. In this study we present our experience in the surgical treatment of disc herniations.

## 1. Introduction

The surgical treatment of lumbar disc herniation is performed when the conservative treatment is recalcitrant and only ten percent of all lumbar disc herniations cases are candidates to surgery [1]. The main problem with the surgery is that the lumbar pain of the patients does not necessarily relieved following surgery and even they might become worse. For this reason, there are serious anxiety and suspicion against the surgical treatment of lumbar disc herniations. This phenomenon is also valid for some spine surgeons who will perform the operation. Even on their own series of Mixter and Barr, who first performed the discectomy of lumbar disc herniations, the success and failure rates compete head to head [2]. Later Caspar and Yasargil introduced the microscope into the disc surgery and allowed minimal anatomic damage; however, no significant rise was achieved in satisfactory results [3, 4].

Carragee et al. revealed that the occurrence of disc herniation, the type of surgery, and the rates of reherniation are in a close relation with the defect on posterior annulus [5]. Lumbar disc herniation is not a separate illness but a part of a degenerative process, so the treatment should be designed in this manner. It is known that if the defect on the annulus is small, annulus has capacity to repair itself after fragmentectomy with both operative techniques: endoscopy and microdiscectomy. On the other hand, if the defect is large, problem arises at that time [6, 7].

In this paper, we discussed our results in the light of literature. We evaluated the role of load sharing principle with application of posterior transpedicular dynamic stabilization (PTDS) in lumbar disc herniation cases with large annulus defect, instead of performing radical discectomy.

## 2. Materials and Methods

**Figure 1:**
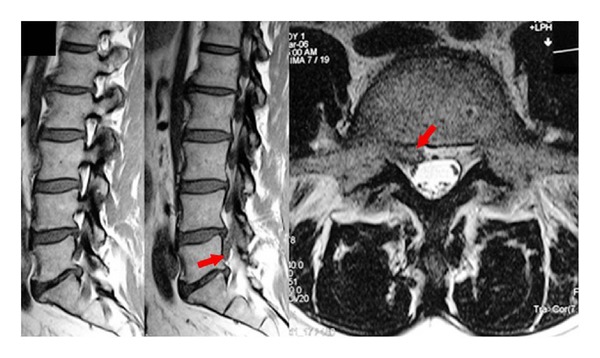
A small extruded fragment was observed under the nerve root. Notice that there is no apparent annulus defect (Carragee Type I).

**Figure 2:**
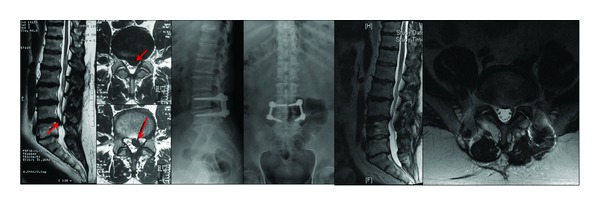
A large extruded fragment and noncontained disc herniation compress right S1 nerve root and cauda equina. Integrity of annulus fibrosus completely destroyed (Carragee Type II). The patient was operated on due to severe neurologic deficit and PTDS was applied to the patient after L5-S1 microdiscectomy and annular repair.

**Figure 3:**
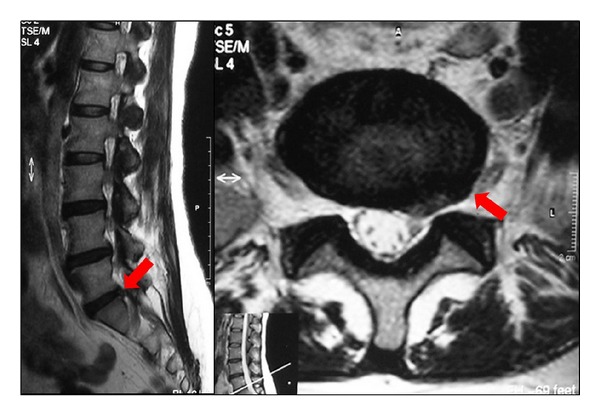
A small annulus defect (<4 mm) was observed at the left side just under the S1 nerve root. Integrity of the annulus fibrosus is preserved (Carragee Type III).

**Figure 4:**
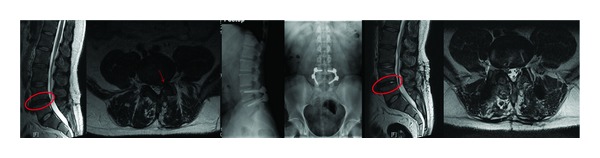
A large annulus defect (>4 mm) was observed at the midline of posterior annulus fibrosus. Integrity of annulus is preserved (Carragee Type IV). The patient is unresponsive to the conservative treatment and PTDS was applied to the patient after L5-S1 microdiscectomy and annular repair.

This is a prospective study held between 2008 and 2012. Totally 98 patients were included in the study who did not respond to conservative treatment and minimal invasive pain procedure that was applied at a minimum of 6 weeks. Conservative treatment includes back exercises program and medicine. Epidural steroid injection and anauloplasty with laser were also performed for some of these cases as a minimal invasive pain procedure. Five surgeons performed the operations. The patients included for the study met the following inclusion criteria: (1) the findings of neurologic examination concordant with the patient's sciatica, (2) one level lumbar disc herniation determined with MR, (3) the surgical procedure applied electively, and (4) not having a spine operation before. Additionally, the patients with infection, instability, scoliosis, and malignancy are excluded from the study. The type of the operation to be applied was told to all the patients and the consent of patients was taken. Before the operation, magnetic resonance imaging was done to all cases and the deformation of annulus was evaluated with MR study and under the surgical microscope in operation. Patients were divided into four groups according to the classification of Carragee et al. [5] with a slight modification. In regard to achieving low recurrence notes, we accepted annulus defect as 4 mm difference from Carragee:

- Type I: there is no significant defect on annulus (Figure 1),
- Type II: annular defect > 4 mm (Figure 2),
- Type III: annular defect < 4 mm (Figure 3),
- Type IV: massive-large annular defect (Figure 4).

The mean age of the patients was 48.19 (between 16 and 80). We determined the type of surgical intervention in reference to CS based on intraoperative observation and MR study. Clinic results were evaluated with visual analog scale (VAS) and Oswestry Disability Index (ODI) in the 3rd, 12th, and 24th months after the surgery. All patients who developed severe low back pain and/or recurrence sciatica were evaluated with MR for recurrence of disc herniation. 

### 2.1. Surgical Technique

The surgical interventions were applied by five surgeons using standard microsurgical techniques at the same hospital. Before the surgical intervention, a single prophylactic antibiotic was given. According to the classification of modified Carragee, only fragmentectomy was applied to the cases of Type I herniation and discectomy was not applied in the course of the operation since annular tear was not observed under the surgical microscope. Limited discectomy was applied to the patients in the other groups. While discectomy was performed through the interlaminar gap in most of the patients, discectomy was applied to some patients following laminotomy with use of high speed drill. In the course of the operation the types of disc herniation were defined with regard to MCC.

In the cases with MC Type II herniation, fragmentectomy (annular tear > 4 mm), limited discectomy with excision of degenerate nucleus pulposus, and annulus repair were performed. Annulus repair is carried out with bipolar cauterization of damaged outer layers of annulus fibrosus under the surgical microscope. PTDS was applied under C-arm scopy through paravertebral muscles as per Wiltse method, Cosmic (Ulrich GmbH & Co. KG, Ulm, Germany) and Safinaz (Medikon, Ankara, Turkey) screw and rods used for PTDS. In the cases with MC Type III herniations, limited discectomy (annular tear < 4 mm) with excision of degenerate nucleus pulposus and annulus repair were performed and PTDS was applied. In the cases with MC Type IV herniations, limited discectomy (massive annular tear) with excision of degenerate nucleus pulposus and annulus repair were performed. Then PTDS was applied.

In the course of operation, annular structure and the size of the annular defect were evaluated by at least two surgeons.

## 3. Results

Totally 13 out of 98 patients, operated with fragmentectomy, limited discectomy applied to 20 patients, and limited discectomy and PTDS were applied to 65 patients. The frequent type of herniation observed in our study was MCC Type II (47.8%) and the frequency of Type I, Type III, and Type IV was 18.3%, 26.5%, and 7.4%, respectively. Intraoperative complication was not observed. In the course of followup, for patients with Type II herniation, one patient developed a screw break and in one patient we observed screw loosening. It was noticed that these two patients were morbid obese. In the postoperative 8th and 12th month, the instrumentation systems were revised. In Type IV group, in one patient screw break was observed following a severe trauma. The instrumentation system of this patient was revised in 16th postoperative month. Finally a recurrence disc herniation was observed in a patient whose body structure was above the normal standards according to her age. In two patients with Type II and one patient with Type IV, adjacent segment degeneration was monitored. On the other hand, these patients did not complain clinically; therefore an extra surgical intervention was not considered. The follow-up period of cases with Type I group, reherniation, and recurrence herniation were not recorded. In two patients with Type II group reherniation, I in two cases with Type III group reherniation, and in one case with Type IV group, and reherniation as recorded.

## 4. Discussion

Even though it is thought that lumbar disc herniation is a separate disease; in fact, the degenerative change of the vertebrae is a part of the process. Following disc degeneration and before the loss of total disc integrity, the disc becomes clinically problematic due to improvement of painful black disc, degenerative spondylolisthesis, or lumbar disc herniation pathologies. That is why its treatment should be with the concepts by which we approach the degenerative process.

Since the beginning of the surgery for the treatment of lumbar disc herniation, the basic aim has been to increase the rate of success of the surgical treatment. Because while in some patients even there was no recurrence or residue herniation radiologically, there is still low back and/or radicular pain and in the other group who had very successful operation, they might have several recurrences on the same level and the same side. It has been thought that the results of the surgery implemented with little anatomic damage by using surgical microscope will affect in a positive way, and some reports supported this method [3, 4]. But the issues occurring after that period. However the later reports showed that the result did not change much in reference to the classic surgery [8, 9]. For a long time period, it is believed that fibrosis is sufical area which is somewhat more in some patients due to an unknown reason, and it is thought that the surgical success would increase if the improvement of fibrosis is prevented and made a big bid for this subject; yet, in the meantime the segmental instability was missed out [10–12]. Following the symptomatic lumbar disc herniation surgery at the height loss on disc space, the relaxation on the facet joint capsule, and ligamentous structures were well-known alternations. After the surgery, the load on the facet joints increases and it may lead to segmental instability [13–15]. For this reason, segmental instability and chronic lumbar pain which improve after the lumbar discectomy cause this type of treatment to which has been used for long years, become disputable [16]. Only the removal of nucleus pulposus is not suitable to stop the segmental degeneration related to the rotational and translational motions [17–19]. Therefore one of the important reasons of the failure of lumbar surgery is segmental instability. Yorimitsu has been following his patients for more than ten years after the disc surgery and concluded that the frequency of the chronic lumbar pain was more in proportion to reherniation based on the height loss on disc space [16].

Segmental instability has been shown with the radiological and clinical findings. These findings may not always support each other [20, 21]. The association of lumbar disc herniation and segmental instability is declared to be 20% in the literature [22]. Kotilainen determined that 22% of the patients developed segmental instability following one level microlumbar discectomy studies and concluded that 29% of them had chronic lumbar pain [23]. Frymoyer signified that on wide based L4-5 disc hernias, there was severe lumbar pain and it is related to degenerative instability [22].

In the surgical treatment of lumbar disc herniation, it is very obvious that disc tried to be taken out; that is to say, radical discectomy does not solve the problem. Although it was realized, disc should not be completely removed. The more the existing disc structure is kept, the better the patient will become after the operation. That is to say, the theory of being respectful of the integrity became the main topic of the conversation with Spengeler who defined limited discectomy in 1990. This concept was improved more, and it is suggested by Williams that the fragment only should be removed and the integrity of the disc should be protected [24, 25].

Williams reported successful clinic results with minimal disc tissue taken out from the disc while they documented 4–9% recurrence rate and 90% clinic success rate [25, 26]. 

Afterwards, in the literature the discussions began if fragmentectomy or discectomy would be better. Wera et al. compared subtotal discectomy and sequestrectomy in the cases of herniation in Type II. They found that while in the events made with subtotal discectomy, reoperation rate was 3.4%, in the events made only fragmentectomy reoperation rate was 21.2%. Consequently, they informed that in the herniation events in Type II, subtotal discectomy would be more suitable [6]. Rogers compared massive discectomy with fragmentectomy in the disc herniations which are ruptured and reported that in the events of fragmentectomy the recurrence rate was 21% which is in a high rate [27]. Mochida et al. compared the clinical and radiological results of the patients who were operated on by percutaneous nucleotomy and standard discectomy. They documented that in the younger people below 40, surgery performed by protected nucleus pulposus, there were better radiological and clinic results [28]. Thomé et al. stated that recurrence rate is higher by microdiscectomy compared to sequestrectomy [13]. Barth et al. compared the two year rates of reherniation with microdiscectomy and microscopic sequestrectomy. They observed 10.5% reherniation rate in the microdiscectomy group and 12.5% in the events of fragmentectomy and concluded that there was no significant difference between these two groups [29]. However even the results of the patients who had fragmentectomy are better; due to high recurrence rates, some surgeons did not give up performing subtotal discectomy [6, 7].

It is Carragee who emphasized that in the treatment of the lumbar disc herniation, the success is related to the defect on the posterior annulus. Carragee et al. reported in their study that for the patients of Type I group (who had small annular defect with fragment), only fragmentectomy was applied. The rate of reherniation and reoperation was 1%. In the group of Type II (fragment defect), the rate of recurrence sciatica was 27.3%, reoperation rate was high like 21.2%. In the group of Type III (fragment-contained), the rate of recurrence was 11.9% and the reoperation rate was 4.8%. In the group of Type IV (non-fragment-contained), reherniation rate was 37.5% and the rate of reoperation was 6.3%. Only fragmentectomy was applied to Type I group; the other groups were operated on by limited discectomy. Although the clinic results in Type I group were satisfactory, for the other groups, it was observed that the rates of reherniation and reoperation were rather high [5].

Therefore, the persistent pain after the operation and the recurrence is related to segmental instability and directly proportional to the integrity of defect in the posterior annulus. In this study, we applied limited discectomy or fragmentectomy to support posterior tension band; appropriate cases are required in respect to the integrity of disc material. We supported the spine with PTDS. The system shares the load applied on to spine thus decreases the load on the anterior column and this might allow disc to repair itself. Despite the fact that for the patients in Type I and Type III, our approach is the same with Carragee, for patients in Type II and Type IV, we used PTDS in addition to decompression. As a result of this, we achieved better VAS and Oswestry results compared to Carragee and Wera. The rates of recurrence for Type II is 5% and in Type IV is 4%. When we review the patients with recurrence, it was determined that one of them had a trauma in earlier time after the operation and the rest of them were those whose height and weight standards were really high according to the standards of society.

Practically if we exclude the patients who are overweight and had trauma, the rate of recurrence will be lower. It is a necessity that for the overweight people in reference to standards, dynamic systems should be designed restoratively.

In conclusion, the concept of the stabilisation of the spine in motion has been developed lately.

There are still many dark spots such as how much it keeps the motion, long term clinic results are unknown; the effect of it on the adjacent segments are unknown. On the other hand, it has an undeniable reality in its clinical success. Dynamic system technology is open to improvement and it is very certain that we will see the breakthroughs. By time, the dynamic screws, dynamic rods, and even those screws will have the flexibility of their body in the course of adaptation to the bone, will be developed. The rigid systems will leave their places to the systems which will be close to the structure of ligaments. Thus, the use of dynamic systems in the treatment of the cases with Type II and Type IV disc herniations would not be an overtreated approach but it is a step directed to the protection of the disc space following discectomy in more physiological conditionsç.
